# The prosurvival activity of ascites against TRAIL is associated with a shorter disease-free interval in patients with ovarian cancer

**DOI:** 10.1186/1757-2215-3-1

**Published:** 2010-01-18

**Authors:** Denis Lane, Isabelle Matte, Claudine Rancourt, Alain Piché

**Affiliations:** 1 Département de Microbiologie et Infectiologie, Faculté de Médecine, Université de Sherbrooke, 3001, 12ième Avenue Nord, Sherbrooke, J1H 5N4, Canada

## Abstract

**Background:**

The production of ascites is a common complication of ovarian cancer. Ascites constitute a unique tumor microenvironment that may affect disease progression. In this context, we recently showed that ovarian cancer ascites may protect tumor cells from TRAIL-induced apoptosis. In this study, we sought to determine whether the prosurvival effect of ascites affects disease-free intervals.

**Methods:**

Peritoneal fluids were obtained from 54 women undergoing intra-abdominal surgery for suspected ovarian cancer (44 cancers and 10 benign diseases). The ability of peritoneal fluids to protect from TRAIL was assessed in the ovarian cancer cell line CaOV3, and IC_50 _were determined. The anti-apoptotic activity of 6 ascites against cisplatin, paclitaxel, doxorubicin, etoposide and vinorelbine was also assessed in CaOV3 cells, and the prosurvival activity of two ascites was assessed in 9 primary ovarian cancer cultures.

**Results:**

Among the 54 peritoneal fluids tested, inhibition of TRAIL cytotoxicity was variable. Fluids originating from ovarian cancer were generally more protective than fluids from non-malignant diseases. Most of the 44 ovarian cancer ascites increased TRAIL IC_50 _and this inhibitory effect did not correlate strongly with the protein concentration in these ascites or the levels of serum CA125, a tumor antigen which is used in the clinic as a marker of tumor burden. The effect of ascites on cisplatin- and paclitaxel-induced cell death was assessed with 4 ascites having inhibitory effect on TRAIL-induced cell death and 2 that do not. The four ascites with prosurvival activity against TRAIL had some inhibitory on cisplatin and/or paclitaxel. Two ovarian cancer ascites, OVC346 and OVC509, also inhibited TRAIL cytotoxicity in 9 primary cultures of ovarian tumor and induced Akt activation in three of these primary cultures. Among a cohort of 35 patients with ascites, a threshold of TRAIL IC_50 _with ascites/IC_50 _without ascites > 2 was associated with shorter disease-free interval.

**Conclusions:**

The prosurvival activity of ascites against TRAIL is associated with shorter disease-free interval, which may be explained, at least in part, by ascites-induced cisplatin/paclitaxel resistance. Our findings suggest that ascites may contain prosurvival factors that protect against TRAIL and chemotherapy and consequently affect disease progression.

## Introduction

Ovarian cancer is the fifth cause of cancer-related deaths in women, the second most common gynecological cancer, and the leading cause of death from gynecological malignancies [1]. Ovarian cancer is lethal because of invasiveness, insidious progression, and rapid development of resistance to chemotherapy. The incidence of ascites in women presenting with ovarian cancer ranges from 45% to 75% depending on the tumor type [2]. This exudative fluid contains ovarian cancer, lymphoid and mesothelial cells. Ascites fluids also harbour growth factors [3,4], bioactive lipids such as lysophosphatidic acid (LPA) [5], cytokines [6,7] and extracellular matrix constituents [8]. Individually, these factors may promote cell growth [4,5,8], invasion [9], and survival [10] suggesting that ascites play an active role in ovarian cancer progression rather than a passive one. We recently demonstrated that some ovarian cancer ascites inhibit TRAIL- and FasL-induced apoptosis *in vitro *[10]. In that study, six ovarian cancer ascites were tested and five out of six inhibited TRAIL-induced cell death, albeit to different degree. Using the COV2 ascites, we showed that the prosurvival activity was dependent upon the activation of Akt [10]. Given the relatively small number of ascites tested in this study, it was difficult to appreciate whether the prosurvival activity against TRAIL is a common property of ascites or whether it is associated with a specific sub-type of ovarian cancer. In addition, the effect of ascites on primary tumor cells and most importantly the clinical significance of the prosurvival activity of ascites have not been assessed.

The extrinsic apoptotic pathway is activated by death receptor ligand stimulation such as TRAIL. TRAIL binds to its death receptors, TRAIL-R1 and -R2 to activate caspase-8 [11-13]. TRAIL may also interact with two decoy receptors (TRAIL-R3 and -R4) that are unable to transduce death signals [14,15]. Upon TRAIL binding, activated TRAIL-R1 and -R2 recruit FADD (Fas-associated death domain). FADD via its death effector domain (DED) recruits procaspases-8/10, which assemble into a DISC (death-inducing signaling complex) [16]. When recruited to the DISC, procaspases-8 is activated through a series of proteolytic cleavages. Active caspase-8 can directly activate procaspase-3 to execute apoptosis (type I cells) or cleave Bid to produce a truncated form (tBid), which induces release of cytochrome C (cyto C) from the mitochondria and leads to procaspase-9 and subsequently procaspase-3 activation (type II cells) [17]. TRAIL holds great promise as an anti-cancer therapy due to its selective apoptosis-inducing action on tumor cells versus normal cells [18]. TRAIL-based therapies are now in phase I/II clinical trials http://www.clinicaltrials.gov but resistance to TRAIL by tumor cells, including ovarian cancer, may limit its therapeutic use [19-21]. Consequently, to fully exploit the potential of TRAIL, it is essential to understand how the tumor microenvironment may impact on the sensitivity of tumor cells to TRAIL.

In this study, we characterized the effect of a large number of peritoneal fluids isolated from women undergoing intra-abdominal surgery for suspected neoplasia for their ability to inhibit TRAIL-induced cell death in the CaOV3 cell line. These ascites originated from various sub-types of ovarian cancer including serous, endometrioid, mucinous and others. We establish that most ovarian cancer ascites have some inhibitory effect on TRAIL-induced cell death. We also evaluated the antiapoptotic effect of two ovarian cancer ascites *in vitro *on primary cultures of ovarian tumor cells established from ascites (n = 8) or tissues (n = 1). The effect of having ascites with prosurvival activity against TRAIL on disease-free intervals in a cohort of 35 patients was determined.

## Materials and methods

### Primary cultures, ascites samples and human subjects

Informed consent was obtained from women that undergone surgery by the gynecologic oncology service at the Centre Hospitalier Universitaire de Sherbrooke for this institutional review board approved protocol. Peritoneal fluids were obtained at the time of initial cytoreductive surgery for all patients. All fluids were supplied by the Banque de tissus et de données of the Réseau de Recherche en Cancer of the Fonds de la Recherche en Santé du Québec. Histopathology and tumor grade were assigned according to International Federation of Gynecology and Obstetrics (FIGO) criteria. Peritoneal fluids were centrifuged at 1000 rpm for 15 min and supernatants were stored at -20°C until assayed for protein content or XTT. Primary tumor cells were isolated as follow: ovarian cancer ascites were centrifuged at 1000 rpm for 15 min and cells were washed twice with OSE medium (Wisent, St-Bruno, Québec, Canada). Cells were then resuspended in OSE medium supplemented with 10% FBS and β-estradiol (10^-8 ^M) and plated into 75 cm^2 ^flasks. All floating cells were removed the next day. All tumor cell samples were used at low passage (< 10). All patients with advanced ovarian cancer in this study were treated with primary cytoreductive surgery followed by platinum-based chemotherapy. Clinical data were obtained from the medical record. The disease-free interval was defined as the interval between the surgery and the date of progression of the disease. Disease progression was defined by CA125 ≥ 2 X nadir value on two occasions, documentation of increase or new lesions or death [22]. The ovarian cancer cell line CaOV3 was obtained from American Type Culture Collection (Manassas, VA) and maintained in DMEM/F12 (Wisent) supplemented with 10% FBS, 2 mM glutamine and antibiotics at 37°C in 5% CO_2_.

### Reagents

Recombinant human TRAIL was purchased from PeproTech. (Rocky Hill, NJ). Anti-Akt, HRP-conjugated anti-mouse and -rabbit antibodies were purchased from Cell Signaling (Beverly, MA). Anti-phospho-Akt (Ser-473) was from Invitrogen (Biosource, Carlsbad, CA). XTT reagent (2,3-bis-(2-methoxy-4-nitro-5-sulfo-phenyl)2H-tetrazolium-5-carboxonilide) was from Invitrogen. Cisplatin, paclitaxel, doxorubicin, vinorelbine and etoposide were obtained from the hospital pharmacy.

### Cell viability assays

Cell viability in the presence or absence of TRAIL or drugs was determined by XTT assay. Briefly, cells were plated at 20,000 cells/well in 96-well plates in complete medium. The next day, cells (confluence 60-70%) were pre-treated for 2 hrs with or without ascites and then treated with human TRAIL or cisplatin and incubated for 48 h. At the termination of the experiment, the culture media was removed and a mixture of PBS and fresh media (without phenol red) containing phenazine methosulfate and XTT was added for 30 min at room temperature. The O.D. was determined using a microplate reader at 450 nm (TecanSunrise, Research Triangle Park, NC). The percentage of cell viability was defined as the relative absorbance of untreated (no TRAIL, no ascites) versus TRAIL/drugs treated cells in the presence or absence of a specific ascites.

### Immunoblot analysis

Cells were harvested and washed with ice-cold PBS. Whole cell extracts were prepared in lysing buffer (glycerol 10%, Triton X-100 1%, TRIS 10 mM pH 7.4, NaCl 100 mM, EGTA 1 mM, EDTA 1 mM, Na_4_P_2_O_7 _20 mM, NaF 1 mM, Na_3_VO_4 _2 mM, SDS 0.1%) containing protease inhibitors (0.1 mM AEBSF, 5 μg/ml pepstatin, 0.5 μg/ml leupeptin and 2 μg/ml aprotinin) and cytosolic proteins were separated by 12% SDS-PAGE gels. Lysates for phosphorylated proteins were done in the presence of phosphatase inhibitors (100 mM sodium fluoride, 100 μM sodium pyrophosphate, 250 μM sodium orthovanadate). Proteins were transferred to PVDF membranes (Roche, Laval, Québec, Canada) by electroblotting, and immunoblot analysis was performed as previously described [20]. All primary antibodies were incubated overnight at 4°C. Proteins were visualized by enhanced chemiluminescence (GE Healthcare, Baie d'Urfé, Québec, Canada). Densitometric quantification of phosphorylated Akt was performed from three separate experiments normalized to total Akt.

### Statistical analysis

Statistical comparisons between two groups were performed using the Student's *t*-test and with ANOVA when comparing the data with more than two treatments groups. Clinical categorical variables were compared between the two groups with Fisher's exact test. The Pearson's correlation coefficient test was used to estimate the correlation between the protein concentrations or the CA125 levels and TRAIL sensitivity. Progression-free disease analysis was compared using Kaplan-Meier curves coupled with the log rank test. For these analyses, the TRAIL IC_50 _with ascites/TRAIL IC_50 _without ascites were group as having a threshold ≥ 2 or < 2 based on median values. Statistical significance was indicated by *P *< 0.05. Statistical analyses were performed with SPSS software (SPSS Inc., Chicago, IL).

## Results

### Effect of ascites on TRAIL sensitivity

**Figure 1:**
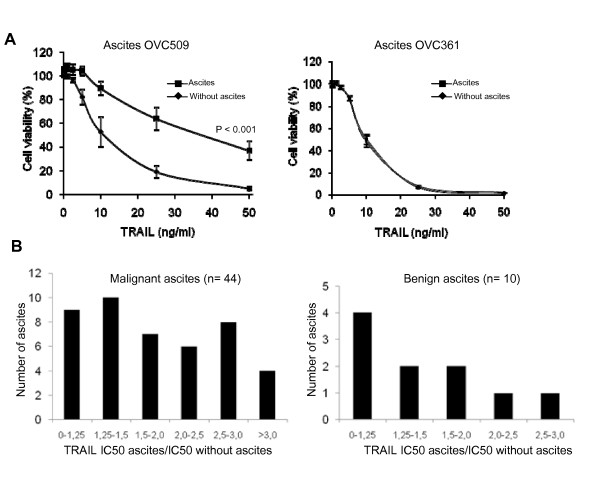
**Effect of peritoneal fluids on TRAIL-induced cell death in CaOV3 cells**. (a) CaOV3 cells were pre-incubated for 2 h with OVC509 and OVC361 ascites (10% v/v) obtained from women with advanced serous ovarian cancer and treated with TRAIL (10 ng/ml) for 48 h. Cell viability was measured by XTT assay. Data are shown as the percent cell viability relative to untreated (no TRAIL, no ascites) cells. Results are from three independent experiments done in triplicate and express as mean ± SEM. (b) TRAIL IC_50 _was determined by XTT assay and defined as the concentration of TRAIL required to kill 50% of CaOV3 cells in the presence or absence of a specific ascites. The prosurvival activity of ovarian cancer ascites and benign fluids was determined by their ability to increase TRAIL IC_50 _after 48 h compared to the TRAIL IC_50 _of CaOV3 cells not exposed to peritoneal fluids. A value of 1 indicates a neutral effect of ascites on TRAIL-induced cytoxicity.

We have previously demonstrated that TRAIL-induced apoptosis was inhibited by the presence of ascites in ovarian cancer cell lines CaOV3 and OVCAR3 as a consequence of Akt activation and up-regulation of c-FLIP_S_, an inhibitor of TRAIL-induced caspase-8 activation [10]. To determine whether the inhibitory effect on TRAIL is a common property of ascites, we analyzed 54 peritoneal fluids. From June 2003 to December 2008, peritoneal fluids from patients undergoing surgery by the gynecologic oncology service at the Centre Hospitalier Universitaire de Sherbrooke for suspected neoplasia were obtained. Tissue biopsies were available for all patients and diseases were classified as benign or malignant according to the histology. To characterize the prosurvival activity of the peritoneal fluids against TRAIL, we assessed the cell viability in the presence or absence of peritoneal fluids at increasing concentrations of TRAIL. Fluids were added to ovarian cancer cell line CaOV3 at 10% of the total assay volume based on our previous study [10]. The characteristics of ascites are shown in Additional file 1, Table S1. Forty four fluids originated from patients with ovarian cancer and 10 were considered benign. Among malignant ascites, most were from patients with serous adenocarcinoma (60%). The protection against TRAIL-induced cell death varied according to peritoneal fluids and examples with OVC509 and OVC 361 ascites are shown in Fig. 1A. OVC509 significantly inhibited TRAIL-induced cell death in CaOV3 cells whereas OVC361 did not. TRAIL IC_50 _was determined from these cell viability curves done with the CaOV3 cell line. The anti-apoptotic activity of ovarian cancer ascites and benign fluids was expressed as TRAIL IC_50 _with ascites/IC_50 _without ascites and is shown in Fig. 1B. Ovarian cancer ascites were generally more protective than fluids from non-malignant diseases (mean IC_50 _increase 2.0 versus 1.25; *P *= 0.02). Most of the 44 ovarian cancer ascites (82%) led to some degree of inhibition of TRAIL-induced apoptosis as demonstrated by an increase of TRAIL IC_50 _with ascites > 1.25 fold while the few remaining did not affect the TRAIL sensitivity of CaOV3 cells (neutral effect). By comparison, 60% of benign fluids displayed an increase of TRAIL IC_50 _> 1.25 fold. It should be noted that we have previously shown that the presence of FBS 10% or conditioned medium from ovarian cancer cells do not affect TRAIL-induced cell death [10]. Furthermore, the anti-apoptotic effect of ascites was almost completely abolished by Akt inhibition in CaOV3 cells [10]. All together, these data demonstrate that most ovarian cancer ascites have an inhibitory effect on TRAIL-induced cell death. The magnitude of this effect however was heterogeneous among ascites. The prosurvival activity of ascites against TRAIL was not associated with a specific tumor sub-type.

### Protein concentration in ascites and serum CA125 levels

**Figure 2:**
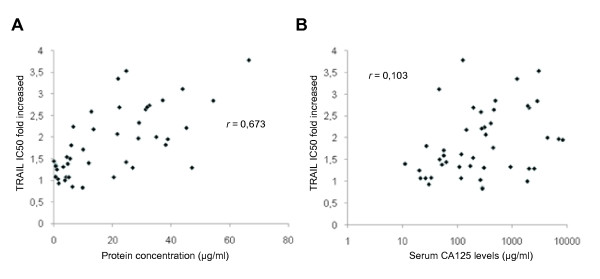
**Protein concentration of peritoneal fluids and baseline serum CA125 levels**. (a) Protein concentration of the 44 ovarian cancer ascites was determined and correlated with TRAIL IC_50 _fold increased mediated by ascites. (b) Baseline serum CA125 levels were obtained for all except one patient and correlated with TRAIL IC_50 _fold increased mediated by ascites. Correlation coefficients (*r*) were determined by Pearson's correlation coefficient test.

The protein concentration was measured in the 54 peritoneal fluids. The mean protein concentration was significantly higher in ovarian cancer ascites than in non-malignant fluids with *P *< 0,001 (data not shown). However, among ovarian cancer ascites, the ability to inhibit TRAIL-induced cell death did not strongly correlate (by Pearson's correlation coefficient test) with the protein content of each ascites (*r *= 0.673; *P *= 0.01) (Fig. 2A).

The CA125 tumor antigen is detected in the majority of serous ovarian carcinoma [23]. It is a mucin-like transmembrane glycoprotein of high molecular weight which is used in the clinic as a marker of tumor burden. There is indeed a strong correlation between rising and falling levels of serum CA125 with progression and regression of the disease [24,25]. CA125 serum levels at presentation reflect to some extent the initial tumor burden. We therefore assessed the baseline serum CA125 levels, which likely reflect the levels in ascites, in our 44 patients with ovarian cancer to determine whether CA125 levels were associated with the anti-apoptotic activity of ascites. As shown in Fig. 2B, the baseline serum CA125 levels did not correlate (*r *= 0.103; *P *= 0,14) with the anti-apoptotic activity of ascites.

### Effect of ascites on drug sensitivity

**Figure 3:**
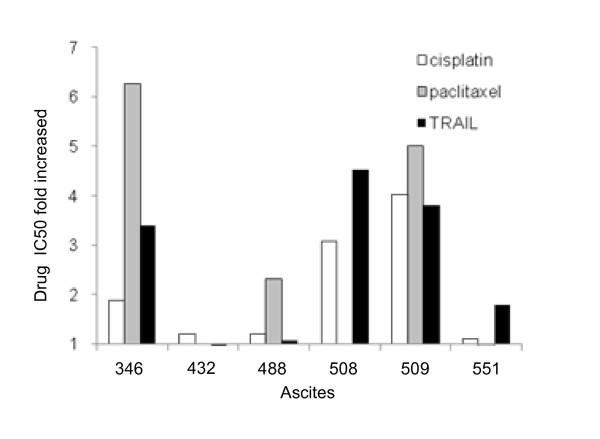
**Effect of ovarian cancer ascites on TRAIL-, cisplatin- and paclitaxel-induced cell death in CaOV3 cells**. CaOV3 cells were pre-incubated for 2 h with various fluids (10% v/v) obtained from women with advanced ovarian cancer and treated with increasing concentrations of TRAIL for 48 h or with cisplatin or paclitaxel for 72 h. Cell viability was assessed by XTT assays. TRAIL, cisplatin and paclitaxel IC_50 _were determined in the presence of ascites and expressed as fold increased relative to IC_50 _in the absence of ascites. A value of 1 indicates a neutral effect of ascites on these drugs. Results are from three independent experiments done in triplicate.

The sensitivity of CaOV3 cells to 5 chemotherapeutic drugs was compared to that of TRAIL in the presence or absence of ascites. Some ascites had anti-apoptotic activity against all drugs (OVC346, OVC509), some against a few drugs only (OVC508, OVC488, OVC551) and some (OVC432) were mostly ineffective (Table 1). All these ascites were obtained from chemotherapy naïve patients (Additional file 1, Table S1). Fig. 3 shows the effect of ascites on TRAIL, cisplatin and paclitaxel-induced cell death, cisplatin and paclitaxel being two drugs that are usually part of the initial treatment for ovarian cancer. Cisplatin IC_50 _was increased by ascites OVC346, OVC508 and OVC509 whereas the other ascites tested had a more limited effect. These three ascites also had an inhibitory on TRAIL-induced cell death. The increase of paclitaxel IC_50 _was observed only with OVC346, OVC488 and OVC509 ascites. Ovarian cancer ascites OVC432 had little anti-apoptotic activity against cisplatin, paclitaxel and TRAIL. These data demonstrate that the inhibitory effect of ascites against drug cytotoxicity is heterogeneous. However, ascites that have a protective effect on TRAIL cytotoxicity are often protective against chemotherapeutic drugs.

**Table 1:** Effect ovarian cancer ascites on drug-induced cell death

Ovarian cancer ascites	Cisplatin IC_50 _(ng/ml)		Paclitaxel IC_50 _(ng/ml)		Doxorubicin IC_50 _(ng/ml)		Etoposide IC_50 _(ng/ml)		Vinorelbine IC_50 _(ng/ml)		TRAIL IC_50 _(ng/ml)	
fluids	-	+	-	+	-	+	-	+	-	+	-	+
**346**	746 ± 38	1400 ± 24	16 ± 7	100 ± 9	86 ± 8	250 ± 15	2183 ± 147	7500 ± 245	4.1 ± 0.25	10 ± 1	8.4 ± 2.7	28.6 ± 4
**432**	746 ± 38	900 ± 43	16 ± 7	16 ± 3	86 ± 8	70 ± 10	2183 ± 147	2000 ± 87	4.1 ± 0.25	4.4 ± 0.4	8.4 ± 2.7	8.1 ± 2.8
**488**	746 ± 38	900 ± 23	16 ± 7	37 ± 5	86 ± 8	107 ± 13	2183 ± 147	6000 ± 184	4.1 ± 0.25	6.3 ± 1	8.4 ± 2.7	9.0 ± 3
**508**	746 ± 38	2300 ± 16	16 ± 7	16 ± 4	86 ± 8	145 ± 5	2183 ± 147	>50000	4.1 ± 0.25	>1000	8.4 ± 2.7	38 ± 4.2
**509**	746 ± 38	3000 ± 54	16 ± 7	80 ± 3	86 ± 8	750 ± 8	2183 ± 147	>50000	4.1 ± 0.25	>1000	8.4 ± 2.7	32 ± 3.1
**551**	746 ± 38	820 ± 47	16 ± 7	13 ± 4	86 ± 8	112 ± 12	2183 ± 147	4600 ± 231	4.1 ± 0.25	6.6 ± 0.2	8.4 ± 2.7	15 ± 5.4

### Ascites decrease TRAIL cytotoxicity in primary cultures of ovarian tumor cells and activate Akt in these cells

**Figure 4:**
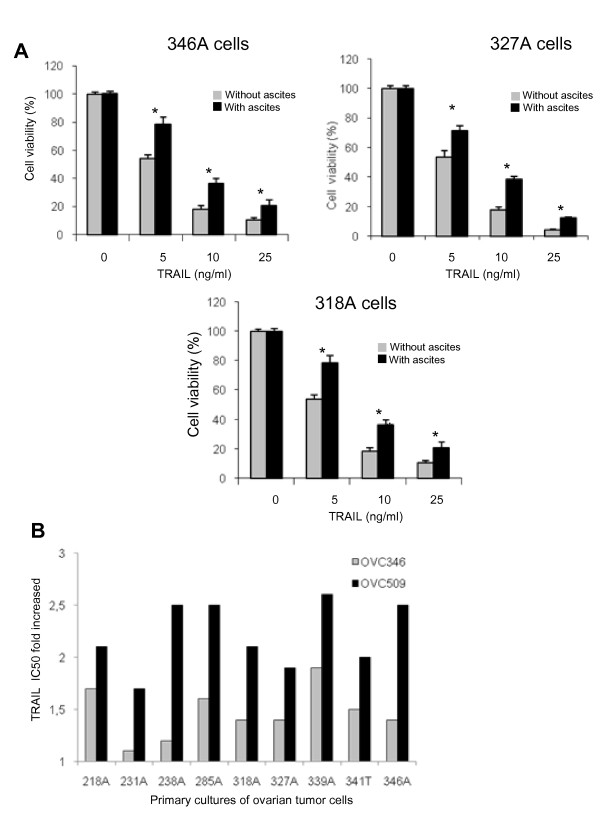
**Effect of ovarian cancer ascites on TRAIL-induced cell death in primary ovarian tumor samples**. (A) Primary cultures ovarian tumor cells (samples 346, 327, 318) were pre-incubated for 2 h with OVC509 (10% v/v) and treated with increasing TRAIL concentrations for 48 h. Cell viability was measured by XTT assay. Data are shown as the percent cell viability relative to TRAIL and ascites untreated cells. Results are from three independent experiments done in triplicate and express as mean ± SEM. *, indicates *P *< 0,001. (b) TRAIL IC_50 _were determined in the presence of OVC346 or OVC509 ascites and expressed as fold increased relative to IC_50 _in the absence of ascites for 9 primary cultures of ovarian tumor cells. Cells were isolated either from ascites (A) or from tissues (T). A value of 1 indicates a neutral effect of ascites on TRAIL cytotoxicity.

The prosurvival activity of ascites against TRAIL cytotoxicity has been shown in ovarian cancer cell lines [10] but has never been demonstrated in primary ovarian cancer cultures. Cell-free ovarian cancer ascites OVC509 were added to primary cultures of tumor cells isolated from ascites obtained from advanced (stage III) serous ovarian cancer patients. TRAIL cytotoxicity was significantly reduced in the presence of OVC509 ascites in primary cultures of tumor cells (346, 327, 318 cells) tested with *P *< 0.001 (Fig. 4A). We extended these data by testing OVC346 and OVC509 ascites in 9 primary cultures. The clinicopathologic data of the 9 primary cultures is shown in Additional file 2, Table S2. TRAIL IC_50 _was determined in the presence or absence of OVC346 and OVC509 ascites in the 9 primary cultures of ovarian tumor cells (Table 2). When expressed as TRAIL IC_50 _fold increased, OVC346 and OVC509 displayed anti-apoptotic activity, albeit at different degree in all 9 primary cultures of ovarian cancer (Fig. 4B). OVC509 had stronger anti-apoptotic activity compared to OVC346.

**Table 2:** Effect ovarian cancer ascites OVC346 and OVC509 on TRAIL IC_50 _in primary samples of ovarian cancer cells

Primary samples	ascites OVC346		ascites OVC509	
ascites	-	+	-	+
**218A**	7.2 ± 1.3	12 ± 1.1	7 ± 0.7	14.5 ± 0.8
**231A**	4.8 ± 0.7	5.5 ± 0.7	4.4 ± 0.8	7.5 ± 0.6
**238A**	16 ± 1.8	19.5 ± 0.9	15 ± 0.4	> 30
**285A**	12 ± 2.1	18.8 ± 1.4	14 ± 1.1	> 30
**318A**	5.5 ± 0.5	7.5 ± 0.7	5.7 ± 0.6	12 ± 0.9
**327A**	5.3 ± 0.7	7.5 ± 0.5	5.2 ± 0.7	9.7 ± 0.8
**339A**	7.8 ± 1.1	14.5 ± 0.7	7.5 ± 1.2	19.5 ± 0.6
**341T**	10.8 ± 1.3	16.5 ± 1.2	10 ± 0.4	19.5 ± 0.9
**346A**	6.5 ± 0.9	9.2 ± 0.6	6.5 ± 0.6	16 ± 1

**Figure 5:**
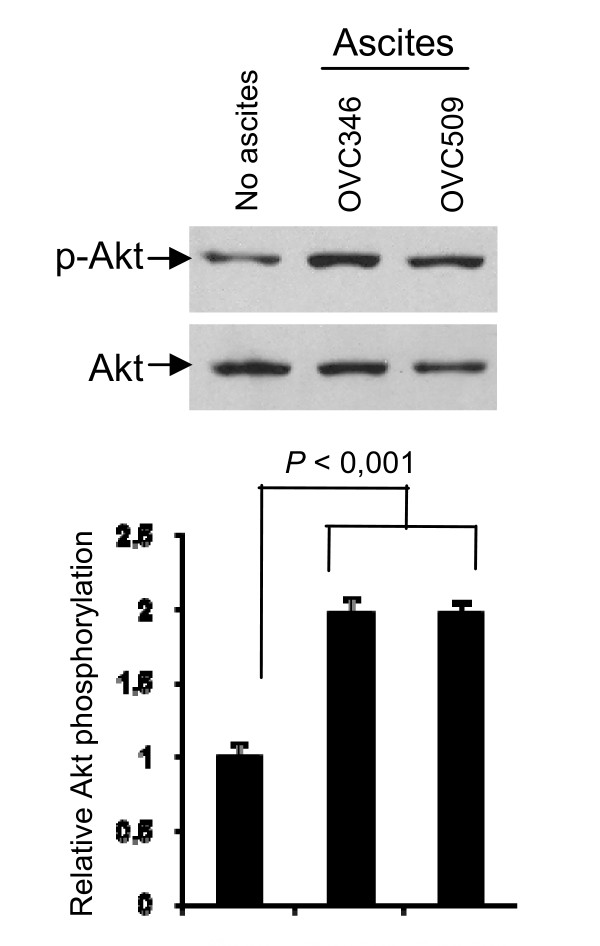
**Ovarian cancer ascites OVC346 and OVC509 were incubated with primary cultures from sample 346A for 90 min**. Lysates were obtained and Western blot analysis was performed with phospho-Ser473 Akt (p-Akt) and Akt antibody (Akt). Densitometric quantification of phosphorylated Akt from three separate experiments normalized to total Akt. Data are expressed as Akt phosphorylation fold increased relative to 349A cells not treated with ascites.

Consistent with our previous findings in CaOV3 cell line [10], we found that both OVC346 and OVC509 ascites induced Akt activation in primary tumor samples as determined by increased Akt phosphorylation on Western blot (Fig. 5). There was a 2 fold increased of Akt phosphorylation mediated by these ascites (*P *< 0.001).

### Prosurvival activity of ovarian cancer ascites and disease-free intervals

**Figure 6:**
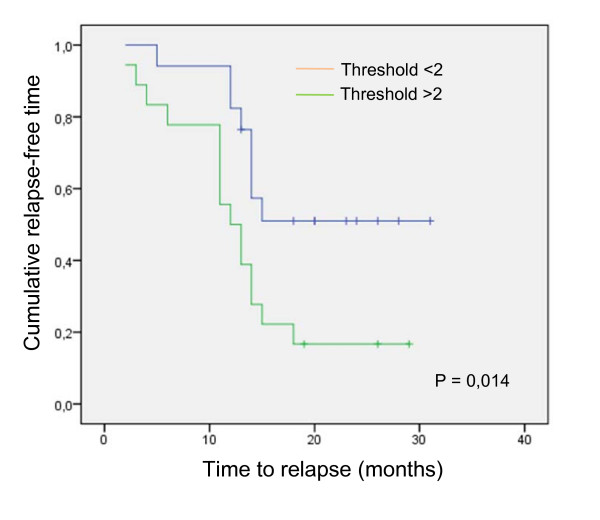
**Impact of having protective ascites on time to first relapse**. Kaplan-Meier curve for 35 patients with ovarian cancer ascites showing the association between protective or non-protective ascites and disease-free interval. Log-rank test was used to verify the significance of the difference (*P *= 0.014).

Among the 44 patients for which we characterized their ascites with regards to TRAIL sensitivity, 35 had follow up > 1 year. We therefore used this cohort of 35 patients to assess the prognosis potential of having protective ascites against TRAIL-induced CaOV3 cell death. Protective ascites were arbitrarily defined as TRAIL IC_50 _with ascites/IC_50 _without ascites > 2-fold. Clinical follow up ranges from 14 months to over 10 years for these 35 patients. The patients were divided into two groups based on whether the ascites isolated from these patients were protective or not against TRAIL-induced cell death. The clinical characteristics of the patients are shown in Table 3. There was no difference between the two groups for age, optimal debulking, tumor histology, stage of disease or grade. Most patients (80%) had advanced disease (stage III or IV). Of note, baseline CA125 levels were similar between the two groups (*P *= 0,064), which suggest that the tumor burden at presentation was not significantly different between the two groups. Kaplan Meier analysis showed that women in the group with TRAIL IC_50 _with ascites/TRAIL IC_50 _without ascites threshold > 2 had significantly shorter time from baseline to first relapse (mean time 12 vs 15 months, *P *= 0.014 log rank) (Fig. 6).

**Table 3:** Baseline characteristics of the patients

Characteristics	Non-protective ascites n = 17	Protective ascites n = 18	*P*
Age (yrs, median)	62 (27-85)	57 (36-88)	NS
Histopathology			
Serous	12 (71%)	15 (83%)	NS
Other	5 (29%)	3 (17%)	
Grade			
1-2	10 (59%)	12 (67%)	NS
3	7 (41%)	6 (33%)	
Stage			
I or II	4 (24%)	3 (17%)	NS
III or IV	13 (76%)	15 (83%)	
Optimal surgery			
Yes	9 (53%)	10 (56%)	NS
No	8 (47%)	8 (44%)	

## Discussion

In this study, using a cell viability-based assay, we evaluated a large number of peritoneal fluids (n = 54) and showed that fluids originating from malignant diseases were generally more protective than fluids from non-malignant diseases against TRAIL-induced cell death. Most of ovarian cancer ascites (82%) led to some degree of inhibition of TRAIL-induced apoptosis as demonstrated by an increase of TRAIL IC_50 _with ascites while the few remaining did not affect the TRAIL sensitivity of CaOV3 cells (neutral effect). The ability of ascites to inhibit TRAIL-induced cell death did not correlate strongly with the protein content of each ascites (*r *= 0.673) or with serum CA125 levels at baseline (*r *= 0.103). Importantly, ovarian cancer ascites also inhibited TRAIL cytotoxicity in primary cultures of tumor cells originating either from ascites (n = 8) or from a metastatic ovarian tumor (n = 1).

We have previously shown that the antiapoptotic activity of ascites was not simply due to the presence of molecules that bind to TRAIL or its receptor and prevent TRAIL binding [10]. Instead, the antiapoptotic activity of ascites was, for the most part, related to the activation of the intracellular survival pathways such as the Akt pathway. The findings that OVC346 and OVC509 ascites activate Akt in primary culture of tumor cells are therefore consistent with our previous observations. Furthermore, proteomic analysis of ovarian cancer ascites demonstrated that malignant cells from ascites have higher levels of activated Akt and discriminated malignant ascites and poor survival outcomes [26]. This is consistent with the fact that PI3K/Akt pathway promotes cell survival by reducing TRAIL-induced apoptosis [10]. The PI3K/Akt pathway is activated in a significant number of ovarian cancers (~70%) and is thought to play an important role in the growth and invasion of ovarian tumors [27]. Activation of this pathway has been associated with cisplatin resistance in ovarian cancer [28]. In addition, the inhibition of Akt prevents the growth of ovarian cancer xenografts [29]. Thus, Akt activation by ascites may promote tumor cell survival and consequently may accelerate relapses.

In CaOV3 cells, although most ascites inhibited TRAIL-induced cell death to some degree, this effect was variable with some ascites increasing TRAIL IC50 by 1.5 to 2-fold whereas others by > 3-fold (Fig. 1B). Furthermore, the specific anti-apoptotic activity of ascites OVC346 and OVC509 differed among primary cultures of ovarian tumor cells (Fig. 4). Similarly, some ascites were effective for inhibiting cisplatin-induced cell death but not paclitaxel-induced cell death and vice versa (Fig. 3). Some were effective to inhibit both drugs. These results suggest that the presence or concentration of prosurvival factors differ in different ovarian cancer ascites. However, ascites that have a protective effect on TRAIL cytotoxicity are often protective against cisplatin. Whether this is related to Akt activation by some ascites in CaOV3 cells is unclear at this point but Akt activation has been associated with the inhibition of cisplatin-induced apoptosis [28].

The present study suggests the importance of ascites as a tumor microenvironment in promoting tumor cell survival. Ovarian cancer is a highly metastatic disease characterized by widespread intraperitoneal dissemination of tumor cells and ascites formation. The intraperitoneal dissemination of ovarian tumor cells involves different processes including migration, survival in peritoneal fluids, invasion and proliferation. Our data show that the prosurvival activity of ascites against TRAIL is associated with a shorter disease-free interval. In previous studies, death receptors or ligands have been reported to be associated with outcome in patients with ovarian cancer. In a study by Conner and Felder the inhibitory effect of ovarian cancer ascites was associated with platinum resistance [30]. Lancaster *et al*. reported that low expression of TRAIL by epithelial ovarian cancer was correlated with a favourable outcome [31]. Several mechanisms underlying the association between ascites inhibitory effect on TRAIL cytotoxicity and shorter disease-free survival may be proposed. Our *in vitro *data demonstrate that the ascites inhibitory effect on TRAIL is often associated with decreased sensitivity to chemotherapeutic drugs. Activation of apoptosis by death receptor ligands is an important mechanism used by the immune system to eliminate floating tumor cells. The functional expression of TRAIL by immune cells in ascites may contribute to the destruction of TRAIL-sensitive cells and limit tumor proliferation and metastasis [32,33]. Inhibition of this process could potentially impact on progression-free survival. Although clinical presentation with stage III or IV and suboptimal surgery are poor prognostic factors, there was no statistical difference between the two groups for these variables. In addition, baseline serum CA125 levels, a surrogate marker for tumor burden, did not correlate with the apoptotic activity of ascites suggesting that the two groups had initial similar tumor burden. Our data raise also the possibility that EOC cells survive in the peritoneal cavity despite active therapy, at least in part, due to the action of anti-apoptotic factors and/or growth factors in ascites that favour tumor cells to re-populate causing tumor relapse.

Our data emphasize the need to continue and expand our understanding of the cross-talk between tumor cells and their microenvironment. The identification of signaling molecules in ovarian cancer ascites and the profiling of activated pathways in tumor cells will be critical for this understanding. Mapping apoptosis-blocking related events may help improve therapies for advanced ovarian cancers.

## Competing interests

The authors declare that they have no competing interests.

## Authors' contributions

AP conceived and designed the study, and drafted the manuscript. Cr participated in substantial contribution in revising the manuscript. DL carried out all *in vitro *studies with ascites. IM performed patient's data collection and ascites samples collection. All authors read and approved the final manuscript.

## Supplementary Material

Additional file 1 **Table S1: Description of ascites samples**. Table S1 describes the characteristics of the 54 peritoneal fluids used in this study. Click here for file

Additional file 2 **Table S1: Clinicopathologic data of primary cultures**. Table S2 describes the characteristics of the 9 primary cultures of ovarian tumor used in the study. Click here for file
