# Supplementary material for: The prosurvival activity of ascites against TRAIL is associated with a shorter disease-free interval in patients with ovarian cancer
Source: J Ovarian Res. 2010 Jan 18;3:1. doi: 10.1186/1757-2215-3-1 (PMC2821314; doi:10.1186/1757-2215-3-1)
Supplement: Additional file 1 — Table S1: Description of ascites samples. Table S1 describes the characteristics of the 54 peritoneal fluids used in this study. [file 1757-2215-3-1-S1.DOC]

| **Ascites** | **Histopathology** | **Grade** | **Stage** | **Prior**  **chemotherapy** | **Minimal**  **Follow up time (months)** | |
| --- | --- | --- | --- | --- | --- | --- |
| COV2 | Serous | 3 | IV | No | | > 60 |
| COV10 | Serous | 3 | IV | No | | > 60 |
| OVC40 | Papillary serous | 3 | IIIC | No | | > 60 |
| OVC41 | Serous | 3 | IV | N/A | | >60 |
| OVC45 | Papillary serous | 3 | N/A | Yes | | >60 |
| OVC48 | Serous | 3 | N/A | No | | >60 |
| OVC346 | Serous | 3 | IIIC | No | | 45 |
| OVC350 | Papillary serous | 3 | IIIC | Yes | | 45 |
| OVC355 | Benign | N/A | N/A | N/A | | N/A |
| OVC361 | Papillary serous | 2 | IIIC | No | | 43 |
| OVC370 | Benign | N/A | N/A | N/A | | N/A |
| OVC373 | Endometrioid | 2 | IIIB | No | | 42 |
| OVC374 | Mucinous | N/A | IC | Yes | | 41 |
| OVC380 | Mucinous | N/A | IA | No | | 40 |
| OVC384 | Benign | N/A | N/A | N/A | | N/A |
| OVC386 | Benign | N/A | N/A | N/A | | N/A |
| OVC395 | Serous | 2 | IV | No | | 37 |
| OVC398 | Endometrioid | 2 | IIIB | No | | 37 |
| OVC401 | Benign | N/A | N/A | N/A | | N/A |
| OVC405 | Serous | 2 | IIIC | No | | 37 |
| OVC409 | Mixed cell | 3 | IIC | No | | 36 |
| OVC410 | Endometrioid | 2 | IIIA | No | | 36 |
| OVC414 | Endometrioid | 1 | IV | No | | 36 |
| OVC415 | Mucinous | 2 | N/A | Yes | | 36 |
| OVC427 | Papillary serous | 2 | IIIC | No | | 34 |
| OVC432 | Mixed cell | 3 | IC | No | | 33 |
| OVC437 | Benign | N/A | N/A | N/A | | N/A |
| OVC439 | Papillary serous | 3 | IIIC | No | | 32 |
| OVC444 | Benign | N/A | N/A | N/A | | N/A |
| OVC445 | Benign | N/A | N/A | N/A | | N/A |
| OVC448 | Mixed cell | 3 | IIIB | Yes | | 30 |
| OVC451 | Mixed cell | 3 | IC | No | | 30 |
| OVC453 | Benign | N/A | N/A | N/A | | N/A |
| OVC461 | Serous | 1 | IA | No | | 28 |
| OVC463 | Papillary serous | 2 | IIIC | Yes | | 28 |
| OVC469 | Papillary serous | 1 | IIIC | No | | 27 |
| OVC472 | Mixed cell | 3 | IIC | No | | 26 |
| OVC483 | Serous | N/A | IA | No | | 25 |
| OVC488 | Papillary serous | 3 | IIIC | No | | 25 |
| OVC489 | Endometrioid | 2 | IIIB | No | | 25 |
| OVC500 | Mixed cell | 1 | IA | No | | 23 |
| OVC503 | Serous | N/A | IV | Yes | | 23 |
| OVC508 | Papillary Serous | 3 | IV | No | | 22 |
| OVC509 | Papillary Serous | 2 | IV | No | | 22 |
| OVC517 | Papillary Serous | 2 | IV | No | | 20 |
| OVC523 | Papillary Serous | 3 | IIIC | No | | 18 |
| OVC530 | Mixed cell | 2 | IIC | No | | 18 |
| OVC535 | Serous | 2 | IV | No | | 18 |
| OVC540 | Benign | N/A | N/A | N/A | | N/A |
| OVC547 | Papillary Serous | 3 | IV | No | | 18 |
| OVC551 | Mixed cell | 3 | IIIC | No | | 18 |
| OVC552 | Papillary Serous | 3 | IIC | No | | 18 |
| OVC563 | Papillary Serous | 3 | IIIC | No | | 12 |
| OVC572 | Serous | 1 | IB | No | | 12 |
