# Supplementary material for: The prosurvival activity of ascites against TRAIL is associated with a shorter disease-free interval in patients with ovarian cancer
Source: J Ovarian Res. 2010 Jan 18;3:1. doi: 10.1186/1757-2215-3-1 (PMC2821314; doi:10.1186/1757-2215-3-1)
Supplement: Additional file 2 — Table S1: Clinicopathologic data of primary cultures. Table S2 describes the characteristics of the 9 primary cultures of ovarian tumor used in the study. [file 1757-2215-3-1-S2.DOC]

| **Primary cultures** | **Age**  **(yrs)** | **Histopathology** | **Grade** | **Stage** | **Prior**  **chemotherapy** |
| --- | --- | --- | --- | --- | --- |
| **218A** | 52 | Normal | N/A | N/A | No |
| **231A** | 56 | Mucinous | N/A | 1C | No |
| **238A** | 55 | Serous | 3 | IIIC | No |
| **285A** | 52 | Serous | 3 | IIIC | No |
| **318A** | 42 | Serous | 2 | IIIC | No |
| **327A** | 61 | Mixed cell | 2 | IIB | No |
| **339A** | 62 | Serous | 1 | IC | No |
| **341T** | 61 | Papillary serous | 3 | IV | Yes |
| **346A** | 76 | Serous | 3 | IIIC | No |
